# Intelligent Point Cloud Processing, Sensing, and Understanding

**DOI:** 10.3390/s24010283

**Published:** 2024-01-03

**Authors:** Miaohui Wang, Guanghui Yue, Jian Xiong, Sukun Tian

**Affiliations:** 1 Guangdong Key Laboratory of Intelligent Information Processing, College of Electronics and Information Engineering, Shenzhen University, Shenzhen 518052, China; 2 School of Biomedical Engineering, Shenzhen University Health Science Center, Shenzhen 518037, China; yueguanghui@szu.edu.cn; 3 School of Communications and Information Engineering, Nanjing University of Post and Telecommunications, Nanjing 210042, China; jxiong@njupt.edu.cn; 4 School of Stomatology, Peking University, Beijing 100081, China

## 1. Introduction

Point clouds are considered one of the fundamental pillars for representing the 3D digital landscape [1], despite the irregular topology between discrete data points. Recent advances in sensor technology [2] that acquire point cloud data to enable flexible and scalable geometric representations have paved the way for the development of new ideas, methodologies, and solutions in ubiquitous sensing and understanding applications. Existing sensor technologies, such as *LiDAR*, *stereo cameras*, and *laser scanners* [3], can be used from a variety of platforms (e.g., satellites, aerial, drones, vehicle-mounted, backpacks, handheld, and static terrestrial) [4,5], viewpoints (e.g., nadir, oblique, and side view) [6], spectra (e.g., multispectral) [7], and granularities (e.g., point density and completeness) [8]. Meanwhile, many promising methods have been developed based on computer vision and deep learning to process the point cloud data [9,10]. However, the expanding applications of point clouds in complex and diverse scenarios, such as autonomous driving [11], robotics [12], augmented reality [13], and urban planning [14], pose new challenges [15] to existing intelligent point cloud approaches.

Recently, artificial intelligence has greatly facilitated the extraction of valuable information from complex point cloud data [16]. Deep learning-based models [16] have shown impressive performance in various point cloud tasks, such as completion [17], compression [18], 3D reconstruction [19], semantic segmentation [19], and object detection [20]. However, as we face increasingly complex and dynamic 3D application scenarios, more accurate, efficient, and effective methods are becoming more and more urgent [21]. Therefore, further investigation on improving intelligent point cloud processing, sensing, and understanding capabilities is of great significance.

This Special Issue collects promising approaches that develop innovative technologies for generating, processing, and analyzing various formats of point cloud data. A total of ten contributions (nine regular articles and one survey) from China, Turkey, Romania, Portugal, the USA, Italy, and the Republic of Korea have been ultimately accepted for publication. These contributions delve into diverse aspects of point clouds, including structural analysis, instance segmentation, registration, texture mapping of 3D meshes, model acceleration and deployment, 3D modeling, up-sampling, plant part segmentation, image-to-point-cloud reconstruction, and LiDAR point cloud (LPC) object detection. The next section provides a concise introduction to each contribution collected in this Special Issue.

## 2. Overview of Contributions

Contribution 1 explored the application of graph kernels in the structural analysis of point clouds, emphasizing their effectiveness in preserving topological structures and enabling machine learning methods on evolving vector data represented as graphs. Specifically, a unique kernel function was introduced to tailor for similarity determination in the point cloud data. To reflect the underlying discrete geometry, the kernel was further formulated based on the proximity of geodesic route distributions in graphs. By demonstrating the effectiveness of the kernel function in supervised classification using a convolutional neural network (CNN), experimental results validated the efficiency of the proposed kernel function for understanding the geometric and topological aspects of 3D point clouds.

Contribution 2 presented a weakly supervised instance segmentation approach for point clouds, addressing the challenge of inaccurate bounding box annotations. To avoid labor-intensive point-level annotations, they first developed a self-distillation architecture that leveraged the consistency regularization, and then utilized data perturbation and historical predictions to enhance generalization, as well as prevent over-fitting to noisy labels. Later, they selected reliable samples and corrected labels based on historical consistency. Experimental results on the benchmark dataset demonstrated the effectiveness and robustness of their approach, achieving comparable performance to existing supervised methods and outperforming recent weakly supervised methods.

Contribution 3 proposed a robust alignment scheme for point clouds, where the rotation and translation coefficients were calculated using the angle of the normal vector of the building facade and the distance between outer endpoints. Experimental results demonstrated the feasibility and robustness of their alignment method on homologous and cross-source point clouds. In addition, they also pointed out that the future work can further optimize the efficiency of parameter-dependent building facade point extraction and explore applications to point cloud registration with varying sensor qualities.

Contribution 4 developed a novel sequential pairwise color-correction approach to mitigate texture seams generated from multiple images. By selecting a reference image and computing the color correction paths through a weighted graph, this approach could effectively enhance the color similarities among different images, resulting in high-quality textured meshes. Experimental results show that the proposed method outperforms existing schemes in both qualitative and quantitative evaluations on an indoor dataset, especially in scenarios with high triangle transitions.

Contribution 5 designed a light-weight CNN model for moving object segmentation in LPCs, addressing the challenge of real-time processing on embedded platforms. The proposed network achieved a reduction in parameters compared to the state of the art, demonstrating efficient processing on the *RTX 3090 GPU*. In addition, it has been also successfully implemented on an FPGA platform, achieving 32 fps for moving-object segmentation, meeting the real-time requirements in autonomous driving. Despite its comparable error performance with significantly fewer parameters, this light-weight model faced potential challenges, such as simplifying the network structure without compromising performance and addressing the sacrifice of low-level details for computational acceleration.

Contribution 6 addressed the challenge of accurately representing cultural heritage objects for finite element analysis (FEA) to understand their mechanical behavior. Unlike the use of traditional CAD 3D models and non-uniform rational B-spline surfaces (NURBS), they employed an alternative method utilizing the re-topology procedure to create simplified yet accurate 3D models for FEA. This study emphasized the importance of retaining the formal definition compatible with FEA software, demonstrating its effectiveness for morphologically complex objects. Experimental results demonstrate that the proposed method can reduce the mesh size, while maintaining high accuracy compared to high-resolution reality-based models. Future work can be developed to improve interoperability, material segmentation, and detailed parameterization for a more comprehensive understanding of the structural behavior of cultural heritage objects.

Contribution 7 proposed a point cloud up-sampling via multi-scale features attention (PU-MFA) method, leveraging the U-Net structure to combine multi-scale features and cross-attention mechanism. PU-MFA was developed to adaptively and effectively use multi-scale features, demonstrating superior performance in generating high-quality dense points. Experimental validations on synthetic and real-scanned datasets show the effectiveness of PU-MFA. It is worth noting that PU-MFA currently has limitations in addressing arbitrary up-sampling ratios.

Contribution 8 introduced the MASPC_Transform, a segmentation network for plant point clouds designed to address the challenges posed by the intricate and small-scale nature of plant organs. Leveraging a multi-head attention separation and a spatially grounded attention separation loss, MASPC_Transform established connections for similar point clouds scattered across different areas in the point cloud space. Additionally, a position-coding method was proposed to enhance the feature extraction in the presence of disordering point clouds. Experimental results demonstrated that MASPC_Transform outperformed existing approaches on the plant segmentation. Finally, they also emphasized the need for further testing on new open-source datasets to validate the generalizability of the MASPC_Transform.

Contribution 9 presented a novel 3D-SSRecNet network for efficient 3D point cloud reconstruction from a single image. 3D-SSRecNet was composed of a 2D image feature extraction network based on a backbone network for object detection, and a point cloud prediction network for minimizing the reconstruction loss. The specially chosen activation function was then employed for better shape prediction and lower reconstructed error. Experimental results on two datasets demonstrated the promising performance of 3D-SSRecNet. Although 3D-SSRecNet can be considered as a computationally effective solution for point cloud reconstruction, future work can be investigated to further improve local reconstruction effects while maintaining computational efficiency.

Contribution 10 provided a comprehensive survey on deep learning-based LiDAR 3D object detection for autonomous driving. It summarized the commonly used feature extraction and processing techniques for LPCs, the coordinate systems in LiDAR object detection, and the stages of autonomous driving. Furthermore, a deep learning-based LPC object detection methods were classified into three categories: projection, voxel, and raw point clouds. They have also conducted in-depth analyses, comparisons, and summaries of the advantages and disadvantages of existing LPC object detection methods. Finally, they pointed out that there are still many open issues in improving model speed and accuracy to achieve real-time processing for level-4 to level-5 autonomous driving.

## 3. Conclusions

This Special Issue serves as a portfolio, bringing together a wide range of contributions that address crucial challenges and advancements in the region of point cloud processing, sensing, and understanding. The selected papers represent a collective endeavor to push the boundaries of point cloud knowledge, offering intelligent solutions to existing challenges, while also unlocking new applications for 3D point clouds. We believe that the above papers will provide valuable insights for researchers and practitioners in this field, stimulating ongoing evolution towards academic and industrial solutions that are not only more accurate, but also more efficient and effective.

## Data Availability

The related datasets can be referred to each contribution in this Editorial.
